# Data for characterization of the pore wetting process of equal-sized granular coals

**DOI:** 10.1016/j.dib.2022.107887

**Published:** 2022-02-03

**Authors:** Yuebing Zhang, Quangui Li, Qianting Hu, Cheng Zhai, Mingyang Song, Jizhao Xu, Yize Deng, Peng Liu, Yong Sun, Jialin Shi, Liangping Hu

**Affiliations:** a State Key Laboratory of Coal Mine Disaster Dynamics and Control, Chongqing University, Chongqing 400044, China; b School of Resources and Safety Engineering, Chongqing University, Chongqing 400044, China; c School of Safety Engineering, China University of Mining and Technology, Xuzhou 221116, China

**Keywords:** Granular coal, Wetting pore size distribution, Contact angle, Pore wetting

## Abstract

In this paper, all measurement and calculation data and their preparation process are presented in detail, which supplements the information published in this co-submission are related to the article “Characterization of the Pore Wetting Process of Equal-Sized Granular Coals based on LF-NMR” [1]. This includes the preparation and component analysis of samples, surface contact angle measurement, analysis of original *T_2_* spectrum and wetting pore size distribution (W-PSD) conversion calculation process. Hence the reader can use the data for their validations and analysis. LF-NMR experiments were conducted for the granular coal pore wetting characterization at the large-diameter MacroMR12-150H-I imaging and analysis system, of Suzhou Niumai Corporation in Jiangsu Province, China. Combined with contact angle measurement, which used the JY-PHb contact angle test instrument, we analyzed the pore wetting process in porous media and its characterization method.

## Specifications Table

**Table:** 

Subject	Energy
Specific subject area	Pore wetting characterization of coal in coalbed methane development
Type of data	Table Figure
How the data were acquired	T_2_ data: the large-diameter MacroMR12-150H-I imaging and analysis system, of Suzhou Niumai Corporation in Jiangsu Province, China. Contact angle: the JY-PHb contact angle test instrument.
Data format	Raw
Description of data collection	All samples were dried for 24 hours at 105° before testing. The transverse relaxation signals of 0.20-0.30 mm spherical glass beads and 0.18-0.25 mm high-rank coal particles were measured at 0 h, 4 h, 8 h, 12 h and 24 h after 1.5 mL water droplets were added, while the surface contact angle of block coal was measured after grinding. T_2_ data can be directly involved in the calculation.
Data source location	Qinshui coalfield, Shanxi, China Xinmi coalfield, Henan, China Songzao coalfield, Chongqing, China
Data accessibility	The complete data set is with this article.
Related research article	Characterization of the Pore Wetting Process of Equal-Sized Granular Coals based on LF-NMR

## Value of the Data

• This article presents pore wetting progress through water dripping on granular coals experiment and LF-NMR measurement. Based on this, the wetting pore size distribution and equivalent wetting pore size characterization method were proposed. These NMR data provide more support for the research paper.
• The NMR raw data and calculation of equivalent wetting pore size is provided here, it would be useful to researchers who are interested in the pore wetting study.
• Understanding the pore wetting process of coal is key to its resource utilization, these data serve as the research basis for relevant researchers to continuously improve the pore wetting characterization method proposed in the research paper.

## 1 Data Description

**Fig. 1:**
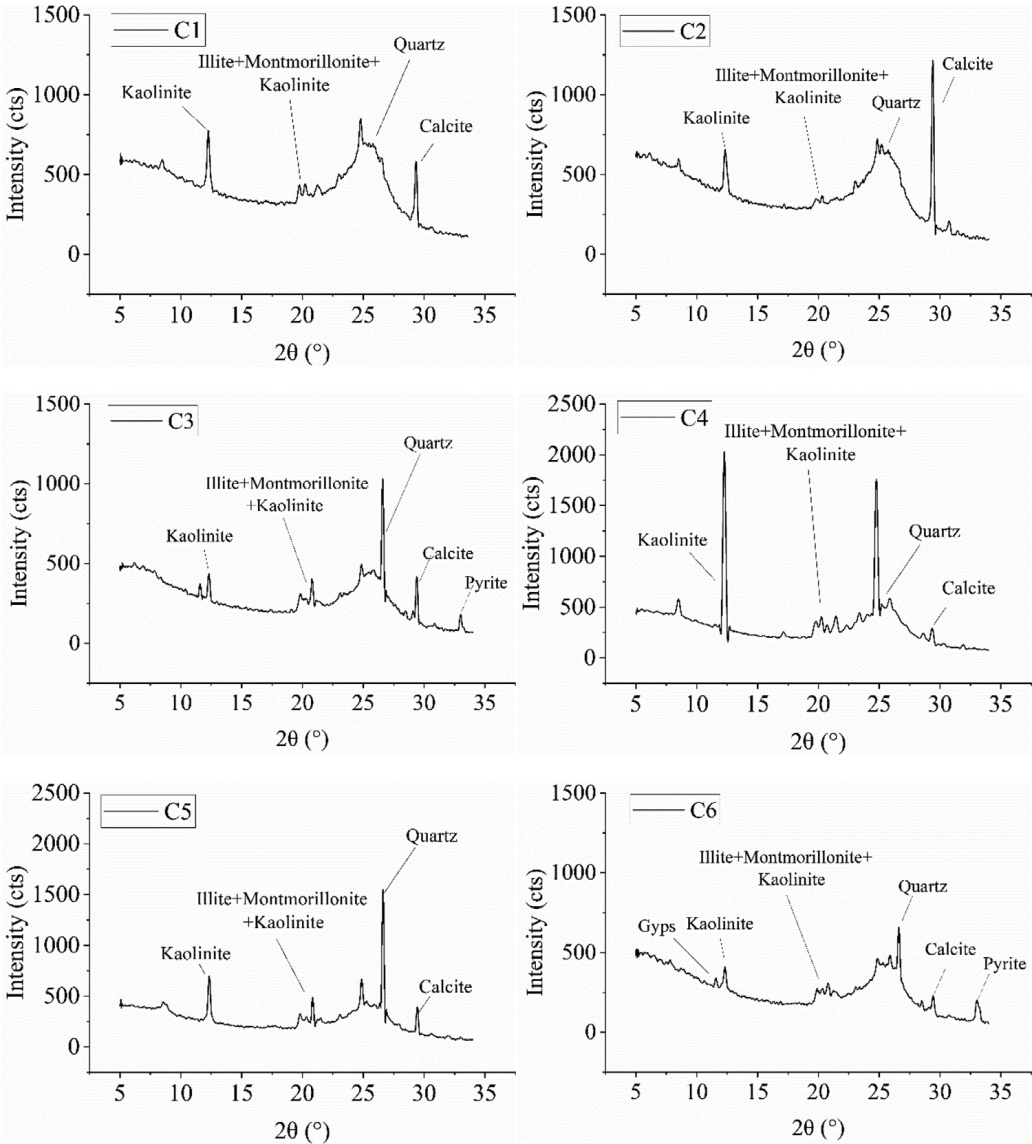
XRD spectrum analysis of each coal sample.

**Fig. 2:**
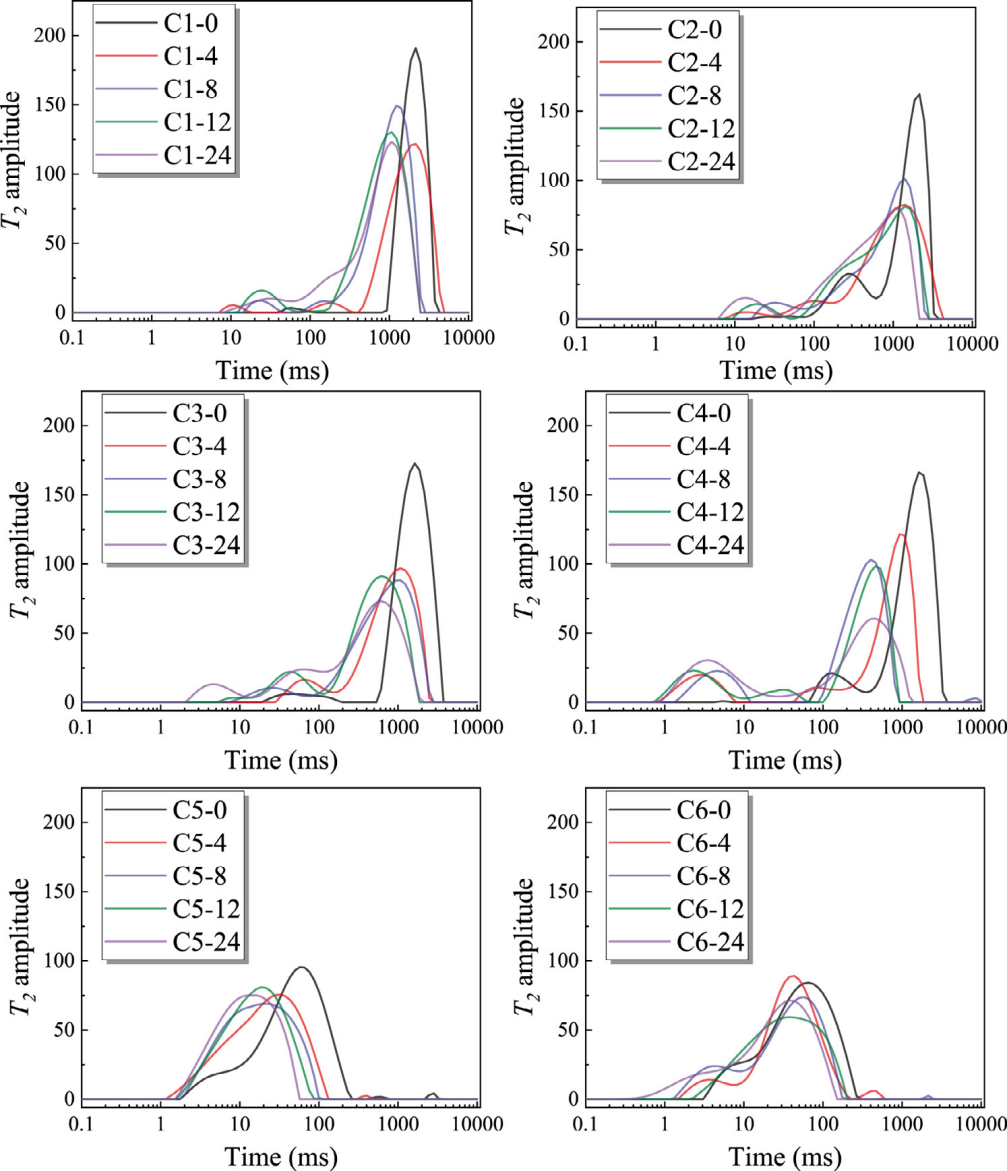
*T_2_* spectrum. (Raw data can be found in Appendix A.)

We present the information of six groups of high-rank coals, including the coal type, proximate analysis, and coalfield, as shown in Table 1. Fig. 1 and Table 2 presents the XRD spectrum of each coal sample and the absolute mineral principal component content of each coal. XRD spectrum was analyzed by X'Pert HighScore software. The mineral content in coal was calculated and compared according to the mineral content is about 1.08 times that of coal ash [2]. The surface contact angle of six groups of coals was measured and recorded in Table 3. NMR original data in different times during pore wetting is shown in Fig. 2, and the original data could be found under the sheet of “Original Data of T_2_” in the supplementary materials. Additionally, other data in the supplementary materials are calculated data of *R_g,h_*.

**Table 1:** Detail information on coal.

Coalfield	Coal mine	Coal type	Coal seam location	Mad %	Aad %	Vad %	FCad %
Xinmi	Gaocheng	Meagre-coal	25#	1.73	13.21	9.47	75.6
	Gaocheng	Meagre-coal	25#	1.47	7.48	11.89	79.17
	Baiping	Meagre-coal	13#	2.65	11.33	10.76	75.28
Qinshui	Sijiazhuang	Anthracite	15#	4.01	16.39	8.83	72.63
Songzao	Songzao	Anthracite	M7	1.96	17.26	8.68	72.12
	Shihao	Anthracite	6#	3.07	27.55	10.62	58.77

**Table 2:** Absolute mineral principal component content of coal.

Sample	Clay	Quartz	Calcite	Pyrite	Gyps
C1	3.4467	6.4843	0.2966	0	0
C2	0.93741	9.72079	2.183	0	0
C3	7.14329	1.73426	0.35623	0.14062	0
C4	5.4882	4.01006	0.03815	0	0
C5	5.20612	5.85107	0.53456	0	0
C6	8.94629	2.13335	0.19498	0.18351	0.02294

**Table 3:** Surface contact angle information. (Modified from Table 1 of [1]).

Coal mine	Samples	Number	Contact angle/°	Average contact angle/°	Note
Gaocheng	C1	1	103.8943	106.5918	no abnormal
		2	103.3361		no abnormal
		3	112.5450		Interference micro-cracks of some point

	C2	1	86.8845	94.3483	no abnormal
		2	100.1510		no abnormal
		3	96.0095		no abnormal

Songzao	C3	1	85.7428	85.2878	no abnormal
		2	82.7087		no abnormal
		3	82.9991		no abnormal
		4	89.7006		no abnormal

Sijiazhuang	C4	1	74.1128	78.0518	no abnormal
		2	81.9908		no abnormal
		3	-		Interference micro-cracks

Baiping	C5	1	72.7088	75.1641	Microfissure
		2	73.1194		Microfissure
		3	86.8256		Microfissure
		4	68.0027		Microfissure

Shihao	C6	1	78.8319	75.4807	Sporadic mineral distribution
		2	76.4361		
		3	71.1742

## 2 Experimental Materials, Design and Methods

The coal sample treatment steps included crushing, screening (60–80 mesh) and drying. Micro glass beads (0.20–0.30 mm) was purchased and its component is SiO_2_≥68.0%, Na_2_O≤14.0% and CaO≥8.0%. All of them were placed in a beaker at 105 °C in a blast drying oven for 24 hours to remove water. And the water used in the droplet granular coal experiment is distilled water. The large-diameter MacroMR12-150H-I imaging and analysis system, Suzhou Niumai Corporation in Jiangsu Province, China, was used for the granular coal pore wetting characterization. A Carr–Purcell–Meiboom–Gill (CPMG) sequence was used to obtain the complete transverse relaxation signal. A NECH of 10000 was chosen with the echo time parameter set to TE=1 ms, TW to 5000 ms through the preliminary adjustment and literature reference [3].

In addition to the water dripping on granular coals experiment, a free-fluid relaxation experiment using distilled water and a wetting experiment with glass beads were each performed.

For water dropping on granular coals and glass beads experiment, prepared plastic bottles (without magnetic interference) with a certain volume of each sample firstly, then gently shake to make the accumulation surface of granular coals or glass beads smooth. 1.5 mL of distilled water was dropped into the sealed test bottle with a syringe, the bottle caps then closed to avoid excessive water evaporation. The moment when the water is just dropped into the device for testing is recorded as 0 h, and the data obtained was the *T_2_* raw data of 0 h, test every 4 hours, and so on until 24 h [4,5]. The *T_2_* spectra (Fig. 2) can be easily obtained from these raw data. By the Eq. (1) of pore size conversion, the *T_2_* spectrum can be converted into the pore size distribution, which is also called the wetting pore size distribution (W-PSD) in this research. Based on the distribution of the W-PSD, the equivalent wetting pore size (*R_g,h_*) can be calculated by Eq. (2), which can be used to characterize the size of the dominant pore size of the wetting area at a certain time. The relevant calculation process and results are presented in Appendix A.

(1) $$R=F_{s}\rho_{2}T_{2}$$

(2) $$R_{g,h}=exp\left[\sum \frac{\ln\left(R_{i}\right)A_{i}}{A_{total}}\right],\left(h=0,4,8,12,24\right)$$

where *R* is the pore radius of the wetting region, $F_{s}$ is the geometric shape factor (2 for cylindrical pores), $R_{i}$ is the wetting pore size, $A_{i}$ is the signal amplitude corresponding to the wetting pore size, *i* is any value in the range of the horizontal axis in the W-PSD spectrum, and $A_{total}$ is the signal amplitude corresponding to the entire pore size distribution.

For the free-fluid relaxation experiment, this is only a supplementary experiment to show that the presence of particle matter has a significant effect on water relaxation time. The experiment only required a drop of 1.5 mL distilled water into an empty plastic bottle to test once.

## Ethics Statements

Not applicable.

## CRediT Author Statement

**Qianting Hu, Quangui Li:** regional geology study, feasibility assess; **Yuebing Zhang, Mingyang Song, Yize Deng, Jialin Shi and Liangping Hu:** collection of coal samples; **Yuebing Zhang, Yize Deng:** analysis of NMR experiments and discussed these data; **Yuebing Zhang:** collected all data and wrote the manuscript; **Cheng Zhai, Jizhao Xu, Peng Liu** and **Yong Sun:** conception of the study and finished final manuscript; **Quangui Li, Qianting Hu:** improved English.

## Declaration of Competing Interest

The authors declare that they have no known competing financial interests or personal relationships that could have appeared to influence the work reported in this paper.

## Appendix B Supplementary materials

Image, application 1

Image, application 2
