# Supplementary material for: Data for characterization of the pore wetting process of equal-sized granular coals
Source: Data Brief. 2022 Feb 3;41:107887. doi: 10.1016/j.dib.2022.107887 (PMC8847833; doi:10.1016/j.dib.2022.107887)
Supplement: Supplementary file 2 [file mmc2.docx]

| **Nomenclature** | | | |
| --- | --- | --- | --- |
| *T* | Telaxation time | 1/*T* | Relaxation rate |
| *T_1_,T_2_* | Longitudinal and Transverse relaxation time | *ρ*_2_ | Surface relaxivity |
| *S* | Surface area | *V* | Pore volume |
| *R* | Wetting region pore radius | *Fs* | Geometric shape factor |
| *C* | Transformation coefficient | *R_i_* | Wetting pore size |
| *A_i_* | Signal amplitude correspond-ing to the wetting pore size | *A_total_* | signal amplitude corre-sponding to the entire pore size distribution |
| *R_g,h_* | The equivalent wetting pore size at the h moment | *R_g,24h_* | The equivalent wetting pore size at the 24 h |
| *R’_g,24h_* | Modified equivalent wetting pore size at the 24 h | *σ* | Surface tension of water |
| Δ*m_clay_*,Δ*ρ* | Clay mineral content and sur-face relaxivity with respect to C4 | *θ* | The cantact angel |
| *f*_c_ | The capillary force |  |  |
| **Abbreviations** | | | |
| LF-NMR | Low-Field Nuclear Magnetic Resonance | | |
| CPMG | Carr–Purcell–Meiboom–Gill | | |
| TW | Waiting time | | |
| TE | Echo time | | |
| NECH | Number of echoes | | |
| W-PSD | Wetting Pore Size Distribution | | |
